# Clinically Silent Gangrenous Cholecystitis: A Case Report of Two-Week Survival Following Spontaneous Gallbladder Perforation Without Intervention

**DOI:** 10.7759/cureus.96101

**Published:** 2025-11-04

**Authors:** Sarah Al-Zaher, German A Almonte Hidalgo

**Affiliations:** 1 Surgical Gastroenterology, William Carey University College of Osteopathic Medicine, Hattiesburg, USA; 2 General Surgery, William Carey University College of Osteopathic Medicine, Hattiesburg, USA; 3 General Surgery, Highland Community Hospital, Picayune, USA

**Keywords:** acute cholecystitis, biliary pathology, case report, contained perforation, gallbladder perforation, gangrenous cholecystitis, laparoscopic cholecystectomy, type ii gallbladder perforation

## Abstract

Spontaneous gallbladder perforation is an infrequent but life-threatening complication of acute cholecystitis, often associated with rapid clinical deterioration and high mortality. Prompt diagnosis and surgical intervention are critical. Atypical presentations in gallbladder perforation, especially if silent, often delay recognition and treatment, increasing the risk of adverse outcomes. We present the case of a 68-year-old male who presented to the emergency department with generalized weakness. Notably, he reported an episode of severe abdominal pain two weeks prior, which had since resolved. Vital signs and physical exam were normal. Imaging demonstrated pneumobilia and a pericholecystic fluid collection consistent with a contained gallbladder perforation. The patient underwent urgent laparoscopic cholecystectomy, which revealed a mid-body gallbladder perforation with extensive gallstone spillage and purulent drainage. Pathology confirmed gangrenous cholecystitis. He recovered uneventfully following surgery and was discharged on postoperative day seven, with no complications noted at his two-week follow-up. This case illustrates a rare presentation of a type II gallbladder perforation that remained clinically silent for two weeks, without signs of peritonitis or systemic compromise. Clinicians must maintain a high index of suspicion for serious intra-abdominal pathology in elderly patients presenting with vague or atypical symptoms, to ensure timely diagnosis and intervention in cases of gallbladder perforation.

## Introduction

Acute cholecystitis is inflammation of the gallbladder, typically caused by gallstone obstruction of the cystic duct [1]. Clinically, acute cholecystitis usually presents with severe right upper quadrant pain, fever, and leukocytosis, and patients often appear ill and tachycardic, particularly after fatty meals [1]. The severity of the condition can range from mild inflammation to severe necrosis and gangrene [2]. Ultrasonography is the initial and most widely used imaging modality, while CT is useful for detecting complications [3]. Hepatobiliary scintigraphy (HIDA) is the most sensitive test and the diagnostic gold standard, though less commonly performed in practice [3]. Although symptoms may spontaneously subside within days, the risk of complications necessitates definitive treatment. Early laparoscopic cholecystectomy, ideally within 72 hours of the diagnosis, remains the first-line treatment and is associated with improved patient outcomes [4].

Approximately 20% of acute cholecystitis cases progress to gangrenous cholecystitis, which carries a heightened risk of sepsis [5]. Among these cases, 10% result in gallbladder perforation, a life-threatening complication associated with high mortality [5]. Gallbladder perforations have been classified by Niemeier into three types: type I (chronic perforation with fistulous communication), type II (localized perforation with abscess formation), and type III (generalized biliary peritonitis due to free spillage into the peritoneal cavity) [6].

We present a case of type II gallbladder perforation that remained clinically silent for two weeks, a presentation rarely documented in the literature. This case report has been prepared in accordance with the SCARE Guidelines for surgical case reports.

## Case presentation

In November 2024, a 68-year-old Caucasian male with a past medical history of gastroesophageal reflux disease and hypertension (well controlled on lisinopril 10mg daily) was admitted after presenting to the emergency department of a rural community hospital with complaints of generalized weakness. He reported that his symptoms began two weeks prior, initially manifesting as achy abdominal pain localized to the right lower quadrant and periumbilical region following a large meal at Thanksgiving dinner. He had experienced intermittent postprandial abdominal pain over the past eight months, but the most severe episode occurred two weeks ago, subsiding the next day. He denied nausea or vomiting but endorsed episodes of diarrhea over the past two weeks and a low-grade fever.

**Figure 1:**
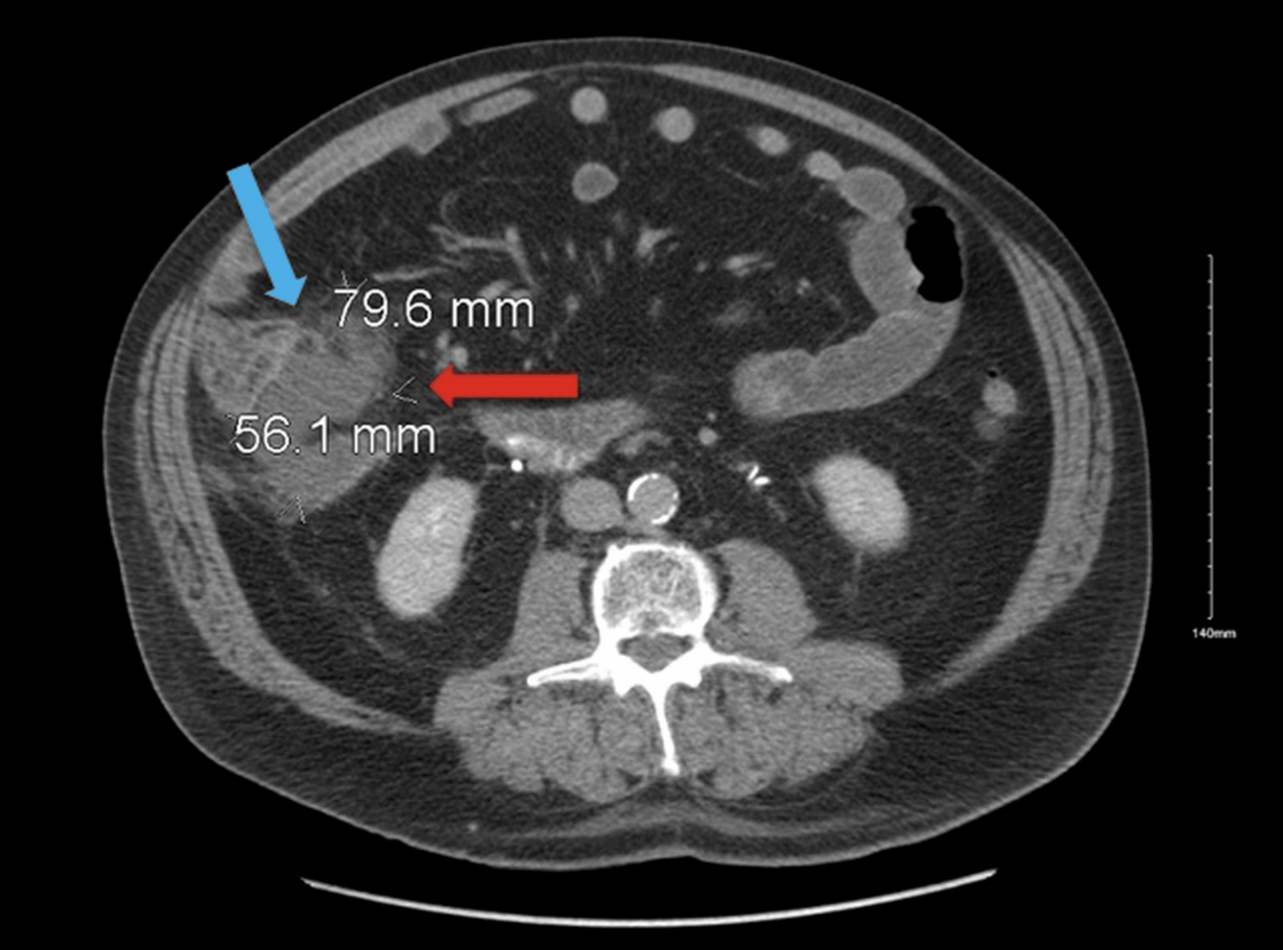
Contrast-enhanced computed tomography (CT) of the abdomen and pelvis (supine view) showing pneumobilia (blue arrow) and a pericholecystic fluid collection (red arrow) consistent with a contained gallbladder perforation. The collection measured approximately 8 × 5.6 cm and extended from the base of the appendix toward the right upper quadrant.

On admission, his vital signs were as follows: blood pressure of 137/54 mmHg, heart rate of 74 beats per minute, respiratory rate of 20 breaths per minute, and a temperature of 37.1°C (98.8°F). During physical examination, the patient denied abdominal pain, with no signs of peritonitis or abdominal guarding. The patient exhibited no signs of distress. Laboratory evaluation revealed leukocytosis with a white blood cell count of 11,600/mm³ (reference range: 4,000-11,000/mm³); other parameters (including liver function tests) remained within normal limits. A computed tomography (CT) scan of the abdomen and pelvis with intravenous contrast revealed air within the gallbladder, a small amount of extraluminal air in Morison’s pouch, and a fluid collection measuring 8 cm x 5.6 cm extending from the base of the appendix toward the right upper quadrant (Figure 1).

**Figure 2:**
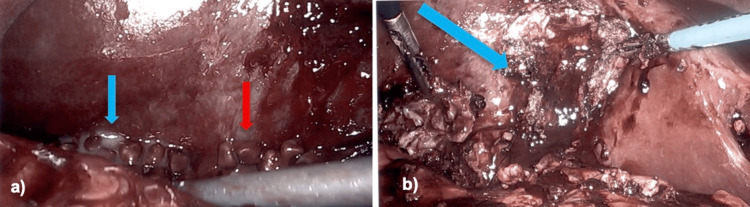
Intraoperative findings during urgent laparoscopic cholecystectomy: (a) perforation at the mid-body of the gallbladder with spilled gallstones (red arrow) and purulent drainage (blue arrow) localized near the hepatic flexure and right colon; (b) fenestrated cholecystectomy specimen (blue arrow) following removal of the gallbladder, with a Jackson-Pratt (JP) drain left in the right upper quadrant for postoperative management.

The preoperative diagnosis was acute cholecystitis with suspected contained perforation. Surgery was performed by a general surgeon with laparoscopic expertise. Intraoperatively, a perforation at the mid-body of the gallbladder was identified, with significant amounts of spilled gallstones and purulent drainage extending to the hepatic flexure and right colon (Figure 2A). Severe inflammation of the hepatocystic plate was also noted. Complete removal of the spilled gallstones, along with thorough peritoneal irrigation lavage, was performed. The gallbladder was removed through a fenestrated cholecystectomy (Figure 2B) and a Jackson-Pratt (JP) drain was placed in the right upper quadrant for postoperative drainage. The patient tolerated the procedure well and remained stable throughout the operation.

Pathological examination of the gallbladder revealed necrotic serosa and an empty lumen, consistent with a final diagnosis of gangrenous acute cholecystitis. Representative histology images were not available for inclusion, which limits the visual documentation of the pathological findings, though the gross and microscopic reports provided sufficient diagnostic confirmation. Intraoperative cultures grew Staphylococcus hominis, which demonstrated sensitivity to multiple agents, including levofloxacin and metronidazole. The patient was started on broad-spectrum intravenous antibiotics, including levofloxacin (750 mg) and metronidazole (500 mg).

The patient had an uneventful recovery. The patient's leukocytosis resolved, his pain steadily improved and his diet was advanced without difficulty. He was discharged on postoperative day seven. At his two-week follow-up in December 2024, the patient was doing well and denied any pain, fever, or gastrointestinal symptoms. His incision sites were clean and well-healed. Repeat laboratory evaluation at that visit showed normalization of leukocytosis, with a WBC of 8,000/mm³. The JP drain, which had been left in situ postoperatively, was removed at this visit without complication. He has since remained asymptomatic, with no readmissions or recurrent biliary symptoms reported. The patient expressed relief that his condition was detected and treated successfully, noting that he had not appreciated the severity of his symptoms until after surgery.

The patient provided written consent for the use of his medical information in the writing of this case report. 

## Discussion

Spontaneous gallbladder perforation is a rare but serious complication of acute cholecystitis, with a reported incidence ranging from 2% to 15% and associated mortality rates as high as 42% in untreated cases [7]. Diagnosis can be challenging due to its overlap in presentation with uncomplicated cholecystitis, often resulting in delayed treatment and increased morbidity [7]. Intraoperative findings in this case - demonstrating a contained, walled-off abscess without evidence of diffuse biliary peritonitis or fistulous communication - are most consistent with a type II perforation. This careful distinction is important, as prognosis and operative considerations differ between localized abscess formation (type II), generalized peritonitis (type III), and fistulous disease (type I) [6].

This distinction is best illustrated by the clinical course of our 68-year-old male patient, with a history of well-controlled gastroesophageal reflux disease and hypertension, who presented with an unusual mild symptom profile - a chief complaint of generalized weakness with no peritoneal signs on physical exam. While he reported chronic postprandial abdominal pain for months, he had never sought care, illustrating the under-recognition of biliary disease in elderly patients. His stable vitals masked the severity of the underlying pathology, illustrating how clinical findings in gallbladder perforation can present as non-specific or subtle. Leukocytosis may be the only laboratory abnormality, and imaging is often the key to diagnosis [8]. Although never formally diagnosed, the patient’s history of postprandial abdominal pain over several months suggests underlying gallbladder pathology, most likely cholecystitis. Risk factors for gangrenous cholecystitis and perforation include advanced age, male gender, diabetes mellitus, cardiovascular disease, and delayed treatment [5]. In this case, advanced age, male sex and hypertension likely contributed to ischemic changes in the gallbladder wall, predisposing the patient to necrosis and eventual perforation.

The patient's clinical presentation and imaging findings strongly suggest that the gallbladder perforation occurred two weeks prior, coinciding with the episode of severe abdominal pain. What makes this case particularly remarkable is the patient's hemodynamically stable presentation and survival without medical or surgical intervention during that period. This is exceedingly rare, as gallbladder perforation is typically associated with rapid clinical deterioration, sepsis, or death if untreated [2].

Derici et al. reviewed 16 cases and found that most patients with gallbladder perforation presented acutely with peritonitis [9]. Type II cases (localized abscess) typically developed within eight to nine days and were associated with abdominal pain, fever, and peritoneal signs, while type I fistulous cases presented after a longer course with bowel obstruction features [9]. Two patients - one with a localized and one with a fistulous perforation - died postoperatively from sepsis and multi-organ failure [9]. Similarly, Abusedera et al. analyzed 20 patients and reported that most localized perforations presented with abdominal pain and fever, with CT playing a critical role in diagnosis and guiding timely surgery [10].

Although type II perforations are typically symptomatic with localized peritonitis, our patient remained virtually asymptomatic and hemodynamically stable for two weeks, an atypical course that underscores the variability of presentation. The absence of fistulous communication and the intraoperative finding of a walled-off abscess without generalized spillage support classification as a type II perforation rather than type III. This containment was likely aided by the omentum and adjacent bowel loops, preventing progression to diffuse peritonitis. Similar to reports by Mehta et al., intra-abdominal abscesses may remain clinically silent or manifest only vague systemic symptoms, particularly in elderly patients, highlighting the importance of early CT imaging when occult perforation is suspected [11]. 

Recent systematic reviews strengthen this context. Quiroga-Garza et al. analyzed 122 patients with type II perforations and found open cholecystectomy reduced subsequent interventions and complications compared to laparoscopic approaches, albeit with longer hospitalization; percutaneous drainage did not alter prognosis [6]. Our case adds to this body of literature by demonstrating that contained perforations can, on rare occasions, remain clinically silent for prolonged periods. In contrast, Quiroga-Garza et al. reviewed another 54 cases of type I fistulous perforations, highlighting their heterogeneity and the need for tailored surgical approaches depending on fistula complexity [12]. By clarifying these patterns, their review emphasizes the importance of distinguishing localized perforations (as in our case) from chronic fistulous disease, since the prognosis and surgical considerations differ significantly.

This case highlights the necessity of maintaining a broad differential diagnosis when evaluating elderly patients with vague abdominal or constitutional symptoms. The absence of classic clinical findings such as fever, right upper quadrant tenderness, or hemodynamic instability does not exclude the presence of significant intra-abdominal pathology. Imaging played a pivotal role in diagnosis. While ultrasonography remains the initial imaging modality for acute cholecystitis, its sensitivity in detecting perforation is limited, especially in atypical presentations [8]. CT is superior for identifying extraluminal air and pericholecystic abscesses, making it the modality of choice in suspected perforation [8]. While HIDA is most sensitive, it is rarely feasible in emergent settings [8]. In patients like ours, early CT can be lifesaving.

This case highlights the necessity of maintaining a broad differential diagnosis when evaluating elderly patients with vague abdominal or constitutional symptoms. The absence of classic clinical findings or hemodynamic instability does not exclude serious intra-abdominal pathology. In this case, CT imaging played a pivotal role in detecting complications that were not apparent on physical examination or routine laboratory evaluation. Although gallbladder perforation is typically associated with rapid clinical deterioration, this case demonstrates that the disease course can be unexpectedly silent for an extended period.

## Conclusions

This case illustrates that gallbladder perforation, though typically associated with rapid clinical decline, can rarely follow an indolent and clinically silent course. Recognition in elderly patients requires vigilance, since subtle or vague symptoms may mask significant pathology. CT remains the most useful modality for early detection when ultrasound findings are inconclusive, and once identified, even contained perforations require urgent surgical intervention. This report is limited by its single-patient nature and short follow-up, which may not capture long-term outcomes. Furthermore, while intraoperative findings supported classification as a type II perforation, overlap with type III features highlights the challenge of strict categorization in clinical practice.

Despite these limitations, several key lessons emerge: (1) Elderly patients with vague or constitutional symptoms should prompt consideration of serious intra-abdominal pathology, (2) CT is the imaging modality of choice for suspected perforation, given the limitations of ultrasound in atypical cases and (3) even clinically silent contained perforations represent surgical emergencies and require timely intervention.
